# Proteasomal degradation of sphingosine kinase 1 and inhibition of dihydroceramide desaturase by the sphingosine kinase inhibitors, SKi or ABC294640, induces growth arrest in androgen-independent LNCaP-AI prostate cancer cells

**DOI:** 10.18632/oncotarget.7693

**Published:** 2016-02-25

**Authors:** Melissa McNaughton, Melissa Pitman, Stuart M. Pitson, Nigel J. Pyne, Susan Pyne

**Affiliations:** ^1^ Strathclyde Institute of Pharmacy and Biomedical Science, University of Strathclyde, Glasgow G4 0RE, UK; ^2^ Centre for Cancer Biology, University of South Australia and SA Pathology, Adelaide SA 5000, Australia

**Keywords:** sphingosine kinase, dihydroceramide desaturase, growth arrest, prostate cancer, sphingosine 1-phosphate

## Abstract

Sphingosine kinases (two isoforms termed SK1 and SK2) catalyse the formation of the bioactive lipid sphingosine 1-phosphate. We demonstrate here that the SK2 inhibitor, ABC294640 (3-(4-chlorophenyl)-adamantane-1-carboxylic acid (pyridin-4-ylmethyl)amide) or the SK1/SK2 inhibitor, SKi (2-(*p*-hydroxyanilino)-4-(*p*-chlorophenyl)thiazole)) induce the proteasomal degradation of SK1a (Mr = 42 kDa) and inhibit DNA synthesis in androgen-independent LNCaP-AI prostate cancer cells. These effects are recapitulated by the dihydroceramide desaturase (Des1) inhibitor, fenretinide. Moreover, SKi or ABC294640 reduce Des1 activity in Jurkat cells and ABC294640 induces the proteasomal degradation of Des1 (Mr = 38 kDa) in LNCaP-AI prostate cancer cells. Furthermore, SKi or ABC294640 or fenretinide increase the expression of the senescence markers, p53 and p21 in LNCaP-AI prostate cancer cells. The siRNA knockdown of SK1 or SK2 failed to increase p53 and p21 expression, but the former did reduce DNA synthesis in LNCaP-AI prostate cancer cells. Moreover, N-acetylcysteine (reactive oxygen species scavenger) blocked the SK inhibitor-induced increase in p21 and p53 expression but had no effect on the proteasomal degradation of SK1a. In addition, siRNA knockdown of Des1 increased p53 expression while a combination of Des1/SK1 siRNA increased the expression of p21. Therefore, Des1 and SK1 participate in regulating LNCaP-AI prostate cancer cell growth and this involves p53/p21-dependent and -independent pathways. Therefore, we propose targeting androgen-independent prostate cancer cells with compounds that affect Des1/SK1 to modulate both *de novo* and sphingolipid rheostat pathways in order to induce growth arrest.

## INTRODUCTION

The bioactive lipid, sphingosine 1-phosphate (S1P) is formed by the phosphorylation of sphingosine and this reaction is catalysed by two isoforms of sphingosine kinase (SK1 and SK2), which are encoded by different genes and exhibit distinct subcellular localisations, biochemical properties and functions (see [1] for review). Once produced, S1P can either be exported from cells (through transporter proteins e.g. *Spns2* and certain ABC transporters) and act as a ligand on a family of five S1P-specific G protein coupled receptors (S1P_1-5_) [2] or, if retained within the cell, bind to and regulate specific intracellular target proteins. For instance, SK2 catalyses the formation of S1P in the nucleus and the subsequently formed S1P inhibits HDAC1/2 activity to induce *c-fos* and *p21* expression [3]. Dephosphorylation of S1P is catalysed by S1P phosphatases that recycle sphingosine, which is then acylated to ceramide. Alternatively, S1P can be irreversibly cleaved by S1P lyase to produce (*E*)-2 hexedecenal and phosphoethanolamine [1].

A feature of SK1 inhibitors is that they induce the ubiquitin-proteasomal degradation of SK1 in solid cancer cell lines [4–6] and proliferating vascular smooth muscle cells [4, 7, 8]. The ubiquitin-proteasomal degradation of SK1 is correlated with binding and/or inhibition of SK1 catalytic activity. However, other inhibitors of SK1 and SK2 such as SKi (2-(*p*-hydroxyanilino)-4-(*p*-chlorophenyl) thiazole) induce the proteasomal degradation of SK1 via a mechanism that is only partially dependent on direct binding to SK1 [4]. Recent studies have demonstrated that SK2 might also have an important role in cancer, For instance, the aryladamantane compound, ABC294640 (3-(4-chlorophenyl)-adamantane-1-carboxylic acid (pyridin-4-ylmethyl)amide), which is reported to be a selective inhibitor of SK2 activity, is in phase 2 clinical trials for diffuse B-cell lymphoma. ABC294640 is a competitive (with sphingosine) inhibitor of SK2 activity with a K_i_ of 9.8 μM, and reduces S1P formation in cancer cells [9]. Oral administration of ABC294640 to mice with mammary adenocarcinoma xenografts results in dose-dependent anti-tumor activity associated with reduced tumor S1P levels and apoptosis [9]. Indeed, ABC294640 also reduces clonogenic survival and viability of ovarian cancer lines and increases caspase cleavage and apoptosis of Kaposi's sarcoma-associated herpes virus positive patient-derived primary effusion lymphoma cells [10]. ABC294640 also promotes autophagic death of A-498 kidney carcinoma cells, PC-3 prostate and MDA-MB-231 breast adenocarcinoma cells [11]. Indeed, ABC294640 delays tumor growth in severe combined immunodeficient mice with A-498 xenografts via an autophagic mechanism. ABC294640 also reduces colorectal cancer growth *in vitro* and *in vivo* [12] and decreases tumor incidence in an azoxymethane (AOM)/dextran sulfate sodium (DSS) mouse model, suggesting that SK2 has a role in inflammation-induced cancer progression [13].

We have previously demonstrated that SKi induces the proteasomal degradation of SK1a and SK1b (which has an 86 amino-acid N-terminal extension compared with SK1a) in androgen-sensitive LNCaP prostate cancer cells and this results in a reduction in S1P levels and an increase in sphingosine and C22:0 and C24:0 ceramide levels. This is associated with the induction of apoptosis [4]. SKi also induces proteasomal degradation of SK1a in androgen-independent LNCaP-AI cells, but fails to reduce SK1b levels [4] and does not increase C22:0 and C24:0 ceramide levels. In this case, androgen-independent LNCaP-AI cells are resistant to apoptosis induced by SKi. Nevertheless, SKi is still able to inhibit DNA synthesis indicative of promoting growth arrest of these cells. The inability of SKi to reduce SK1b expression levels appears due to a compensatory increase in SK1b mRNA expression in these cells. Thus, combined treatment with SK1 siRNA (to prevent mRNA translation of SK1a and significantly, SK1b) and SKi results in apoptosis of androgen-independent LNCaP-AI cells [4].

We have therefore investigated the role of SK1 and SK2 in androgen-independent LNCaP-AI cell growth using the SK1/2 inhibitor, SKi and the SK2 selective inhibitor ABC294640. Our findings indicate that these compounds induce growth arrest predominantly by inducing the proteasomal degradation of SK1 and by inhibiting dihydroceramide desaturase (Des1), which catalyses the conversion of dihydroceramide to ceramide. Thus, growth arrest appears to involve modulation of both the *de novo* ceramide pathway and sphingolipid rheostat (relative effects of ceramide/S1P) pathways.

## RESULTS

### ABC294640 induces the proteasomal degradation of SK1: Reversal by MG132

**Figure 1:**
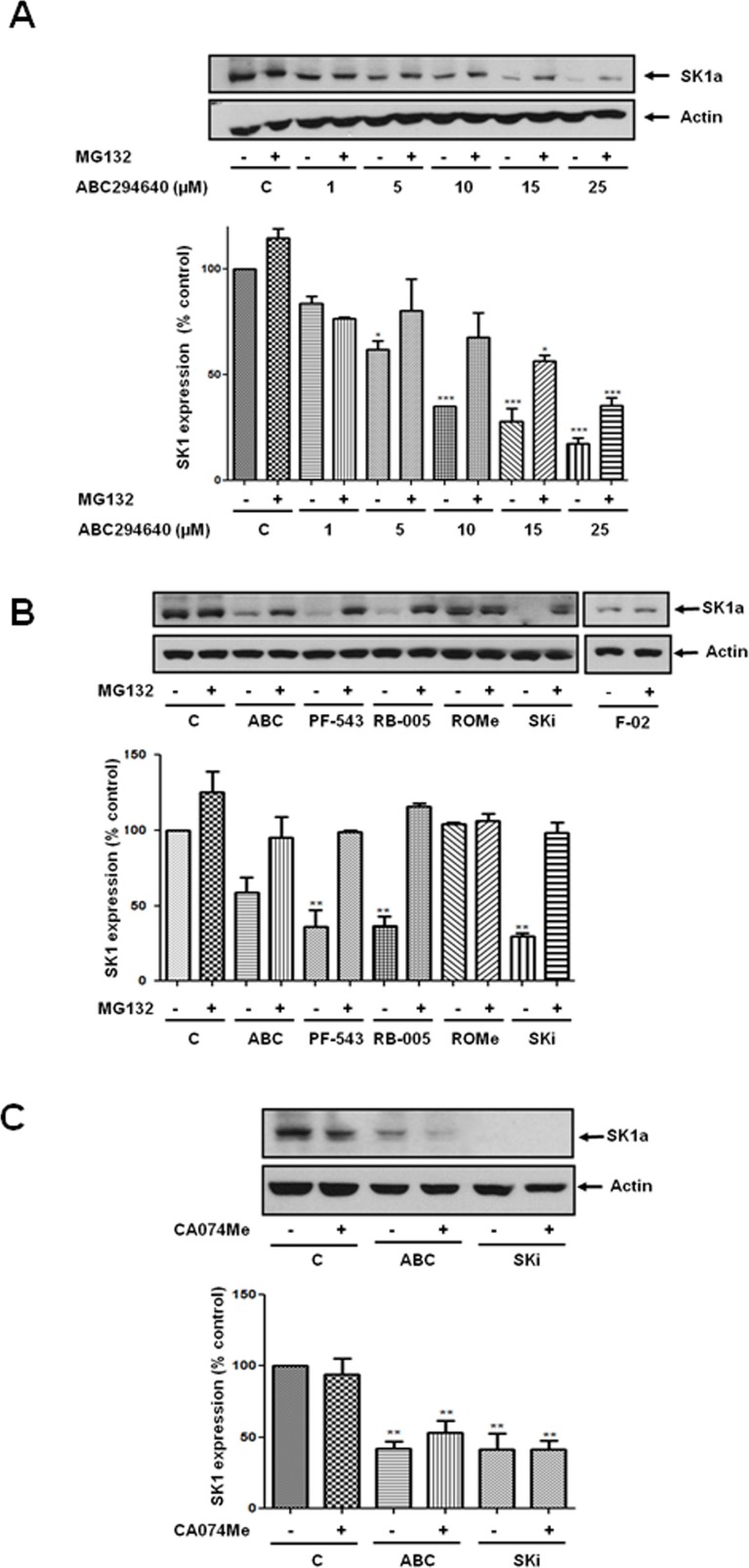

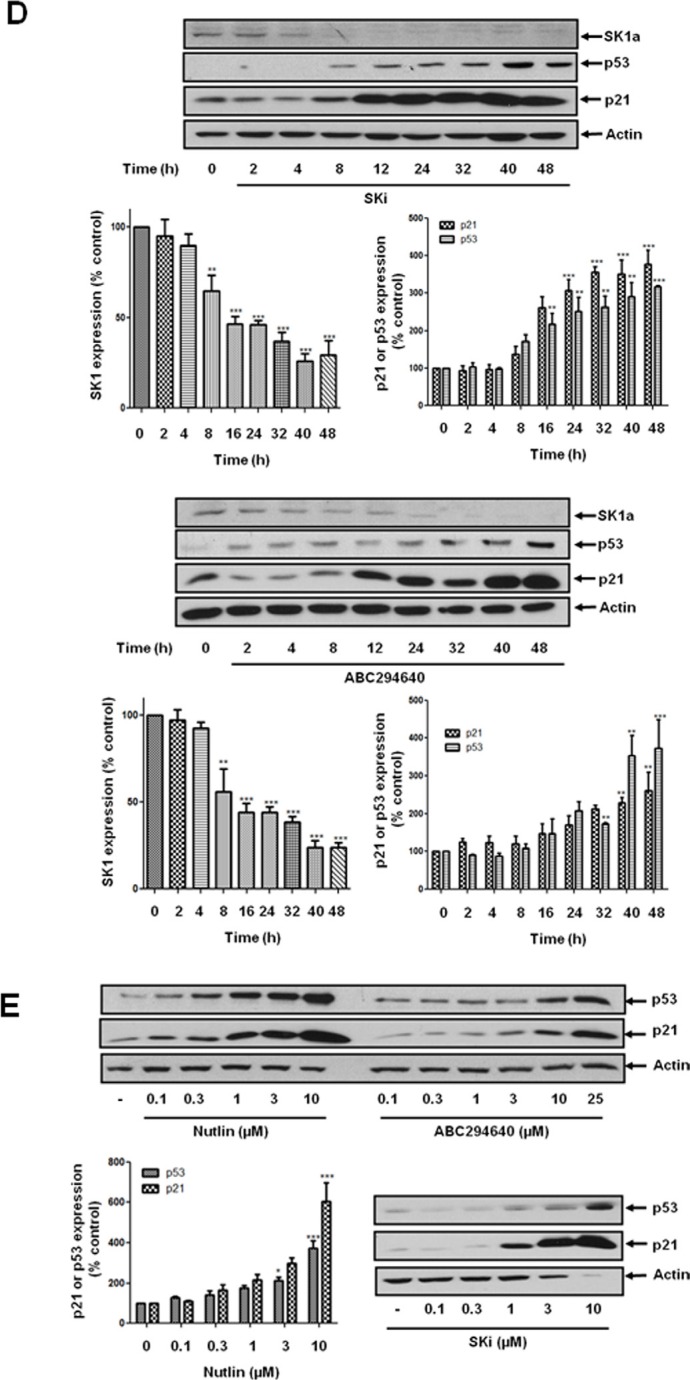
Effect of various SK inhibitors on the proteasomal degradation of SK1a in androgen-independent LNCaP-AI cells LNCaP-AI cells were pre-treated with or without MG132 (10 μM) or CA074Me (10 μM) for 30 min prior to treatment with SK inhibitors for 24 h. (**A**) Western blot showing the effect of ABC294640 (1–25 μM) on the expression of SK1a in the presence and absence of MG132. Also shown is a bar graph of the quantification of the effect of ABC294640 (1–25 μM) on the proteasomal degradation of SK1a. Results are expressed as means +/− SD for *n* = 3 experiments. *p < 0.05, ***p < 0.001 *versus* control; (**B**) Western blot showing the effect of ABC294640 (25 μM) or SKi (10 μM) or PF-543 (100 nM) or RB-005 (10 μM), or F-02 (10 μM) or ROMe (10 μM) in the presence or absence of MG132 on the expression of SK1a. Also shown is a bar graph of the quantification of the effect of SK1- and SK2-selective inhibitors on the proteasomal degradation of SK1a. Results are expressed as means +/− SD for *n* = 3 experiments. **p < 0.01 *versus* control; (**C**) Western blot showing the lack of effect of CA074Me on the ABC294640-(25 μM) or SKi-(10 μM) induced reduction in SK1a expression. Also shown is a bar graph of the quantification of the effect of CA074Me (10 μM) on the SKi-(10 μM) or ABC294640-(25 μM) induced degradation of SK1a. Results are expressed as means +/− SD for *n* = 3 experiments. **p < 0.01 *versus* control; (**D**) Western blot showing the time-course of ABC294640-(25 μM) or SKi-(10 μM) induced changes in SK1a, p53 and p21 expression; (**E**) Western blot showing the concentration-dependence of ABC294640- or SKi- or nutlin on p53 and p21 expression. Also shown in (D) and (E) are bar graphs of the quantification of the effect of nutlin or ABC294640 or SKi on the increase in p21, p53 and decrease in SK1a expression. Results are expressed as means +/− SD for *n* = 3 experiments. *p < 0.05, **p < 0.01 *versus* control. Actin was used a protein loading control in western blots. Western blot results are representative of at least three independent experiments.

Recently, the SK2 selective inhibitor, ABC294640 was demonstrated to induce proteasomal degradation of c-Myc and myeloid cell leukemia 1 (Mcl-1) in multiple myeloma cells [14]. We had previously demonstrated that the SK1/2 inhibitor, SKi also induced the proteasomal degradation of c-Myc in LNCaP prostate cancer cells [15]. In contrast, the SK2 selective inhibitor ((*R*)-FTY720 methylether (ROMe [16]) failed to modulate c-Myc expression in these cells [15]. Therefore, we considered that the ability of ABC294640 to induce proteasomal degradation of c-Myc is not a typical property of SK2 inhibitors but which might be mediated through an indirect effect on the ubiquitin-proteasomal degradation of SK1a in androgen-independent LNCaP-AI cells. Indeed, we show here that ABC294640 (5–25 μM) reduced the expression of SK1a (Mr = 42 kDa) in androgen-independent LNCaP-AI cells and this was prevented by pre-treating these cells with the proteasomal inhibitor, MG132 (Figure 1A). Therefore, although ABC294640 is an SK2 selective inhibitor with no direct inhibitor effect against recombinant SK1 at concentrations as high as 100 μM [9], it is able to remove SK1a from prostate cancer cells *via* an indirect mechanism. These findings were similar to those obtained with the dual SK1/SK2 inhibitor, SKi, which can activate the proteasome and promote accelerated ubiquitin-proteasomal degradation of SK1a in androgen-sensitive and androgen independent LNCaP prostate cancer cells [4]. We therefore, tested the effect of various SK1- and SK2-selective inhibitors on the proteasomal degradation of SK1a in order to establish whether the inhibition of SK2 activity by ABC294640 is required to induce the proteasomal degradation of SK1a. In this regard, the SK1 selective inhibitors PF-543 [17], (which we have shown inhibits SK1 activity with a K_i_ = 14 nM [8] and inhibits SK2 activity by 33% at 5 μM PF-543) and RB-005 (which inhibits SK1 with a K_i_ = 3 μM and inhibits SK2 activity by < 10% at 50 μM RB-005 [7]) induced the proteasomal degradation of SK1a in LNCaP-AI cells (Figure 1B). However, treatment of LNCaP-AI cells with the SK2 selective inhibitors (*R*)-FTY720 methylether (ROMe) (which has a K_i_ = 14 μM, and does not inhibit SK1 activity at 100 μM ROMe [16]) or F-02 [8] failed to reduce SK1a expression in these cells (Figure 1B). We have previously shown that ROMe affects the metabolome of LNCaP cells at this concentration and is therefore active in these cells [15]. We confirm here that the treatment of LNCaP-AI cells with SKi (10 μM) induced a decrease in the expression of SK1a and this could be reversed by pretreatment of the cells with MG132 (Figure 1B).

**Figure 2:**
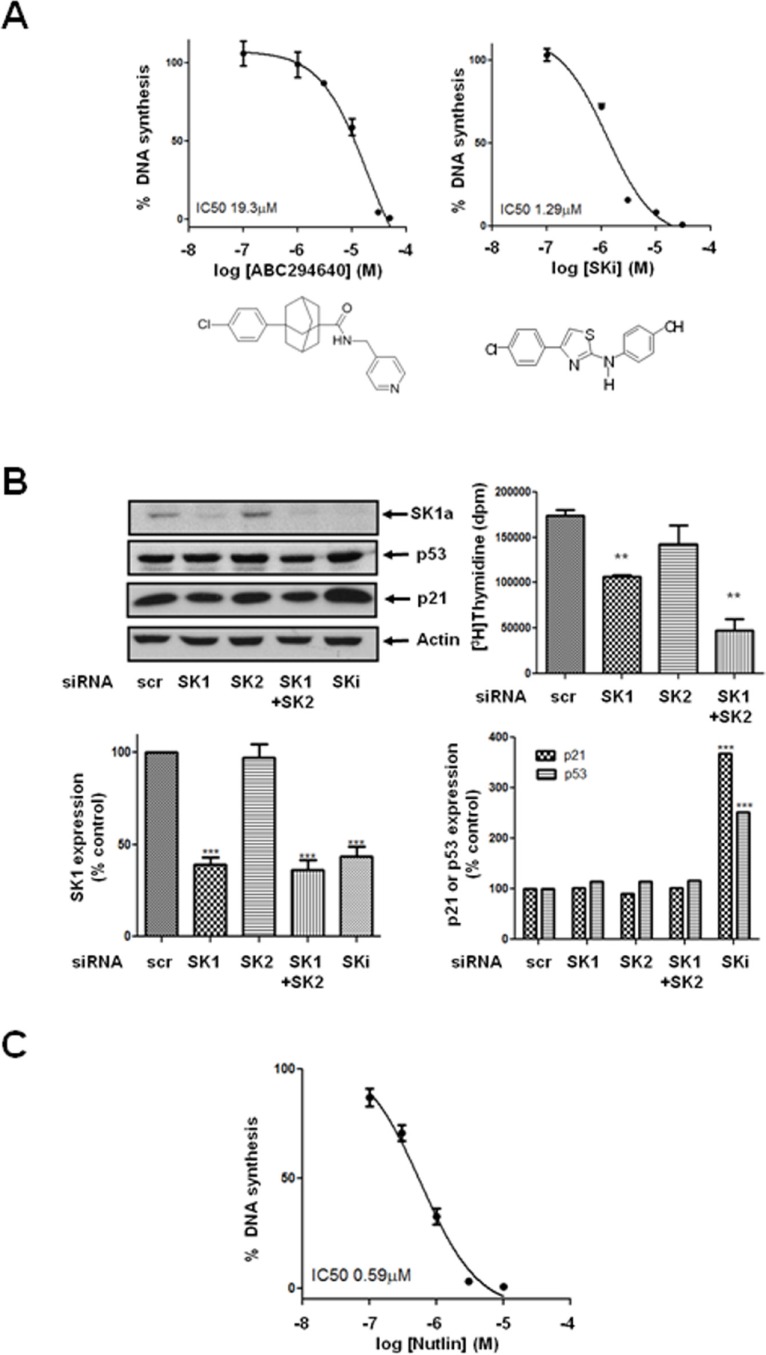
Effect of ABC294640, SKi, nutlin or siRNA knock down of SK isoforms on [^3^H]thymidine incorporation in androgen-independent LNCaP-AI cells Androgen-independent LNCaP-AI cells were treated with ABC294640 or SKi or nutlin for 24 h or scrambled siRNA or SK1 siRNA or SK2 siRNA for 48 h. (**A**) Concentration-dependent inhibition of DNA synthesis is induced by ABC294640 or SKi. Results are expressed as means +/− SD for *n* = 3 experiments; (**B**) Western blot showing the effect of SK1 siRNA or SK2 siRNA on SK1a, p53 and p21 expression. SKi (10 μM) was used a positive control. Actin was used a protein loading control. Blots are representative of three independent experiments. Also shown are bar graphs of the quantification of the effect of SK1 siRNA or SK2 siRNA on SK1a, p53 and p21 expression. Results are expressed as means +/− SD for *n* = 3 experiments. ***p < 0.001 *versus* scrambled siRNA. The effect of SK1 siRNA or SK2 siRNA on DNA synthesis is also shown. **p < 0.01 *versus* scrambled siRNA; (**C**) Concentration-dependent inhibition of DNA synthesis is induced by nutlin. Results are expressed as means +/− SD for *n* = 3 experiments.

Previous reports have demonstrated that SKi can also induce the lysosomal degradation of SK1 by cathepsin B1 [18]. However, pre-treatment of LNCaP-AI cells with the cathepsin B1 inhibitor, CA074Me had no effect on the ability of ABC294640 or SKi to induce the decrease in expression of SK1a (Figure 1C). The effects of SKi or ABC294640 on the proteasomal degradation of SK1a were time-dependent (Figure 1D) and associated with a significant concentration-dependent inhibition of DNA synthesis, and which was recapitulated by siRNA knockdown of SK1 but not SK2 (Figure 2A, 2B).

### Effect on p53 and p21 expression

Treatment of LNCaP-AI cells with ABC294640 or SKi induced a concentration- and time-dependent increase in p53 and p21 expression (Figure 1D, 1E), which are markers of growth arrest and senescence, in androgen-independent LNCaP-AI cells. This was fully recapitulated with nutlin (Figure 1E), an inhibitor of the E3 ligase, mdm2 which catalyses ubquitination of p53 and regulates its stability in cells. In common with SKi and ABC294640, nutlin induced a concentration-dependent inhibition of DNA synthesis (Figure 2C) consistent with a p53/p21-dependent growth arrest. In contrast, the SK1 selective inhibitor, PF-543 and the SK2 selective inhibitor, ROMe failed to induce an increase in p53 expression (Supplementary Figure 1). An individual role for SK1 and SK2 in regulating p53/p21 expression was excluded based on the finding that siRNA knockdown of either kinase or both together had no effect on p53 or p21 expression (Figure 2B).

### The role of dihydroceramide desaturase

**Figure 3:**
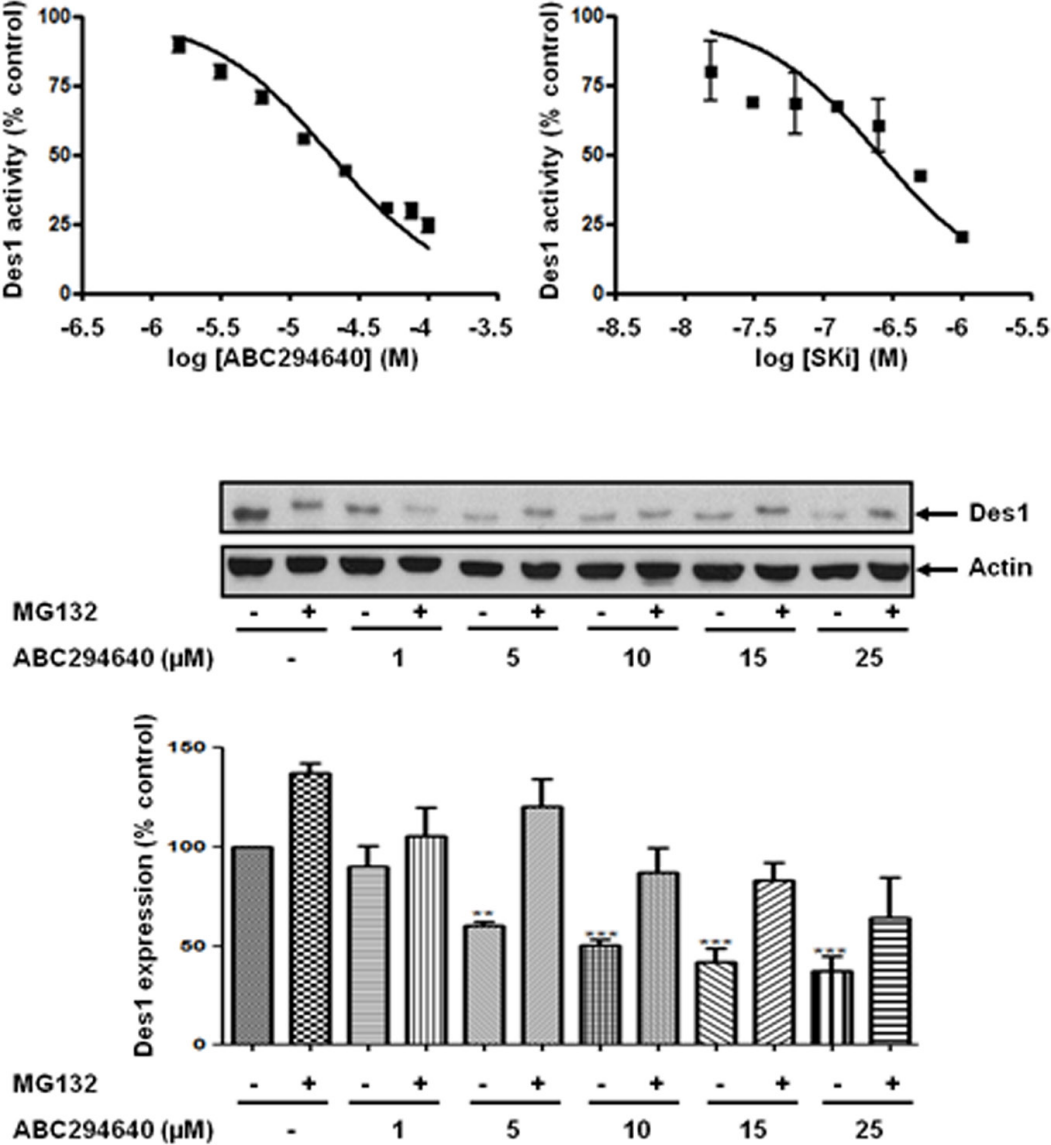
Effect of ABC294640 or SKi on Des1 activity Des1 activity was measured using Jurkat cells labeled with DhCer-C6-NBD and HPLC analysis. The western blot demonstrates the effect of ABC294640 (25 μM) for 24 h in the presence and absence of MG132 (10 μM) on Des1 expression. Actin was used a protein loading control. Results are representative of at least three independent experiments. Also shown is a bar graph of the quantification of the effect of ABC294640 on Des1 expression. Results are expressed as means +/− SD for *n* = 3 experiments. **p < 0.01 *versus* control; ***p < 0.001 *versus* control.

**Figure 4:**
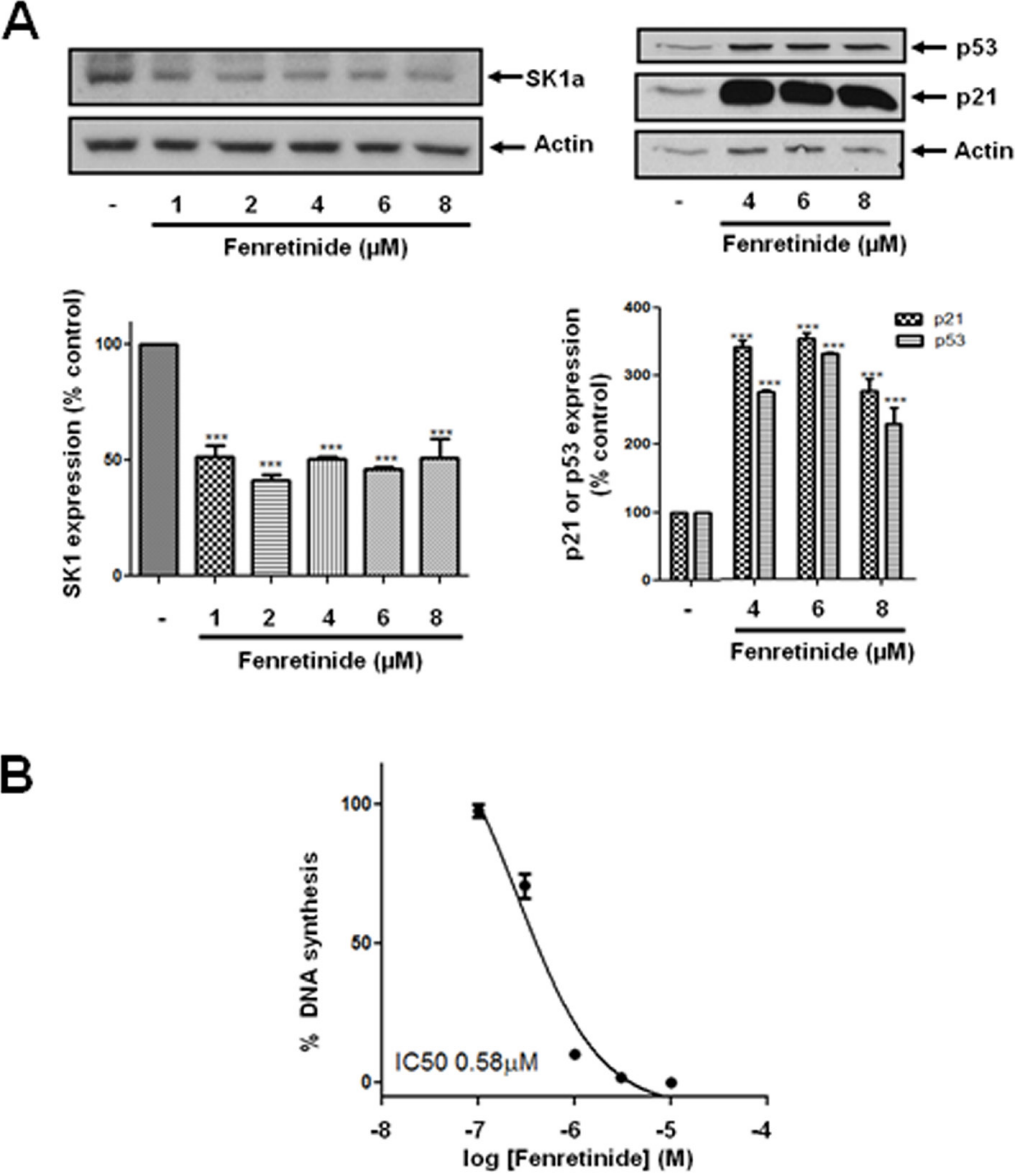
Effect of fenretinide on SK1a, p21 and p53 expression LNCaP-AI cells were treated with fenretinide for 24 h. (**A**) Western blot showing the effect of fenretinide (1–8 μM) on the expression of SK1a, p21 and p53. Results are representative of at least three independent experiments. Actin was used a protein loading control. Also shown are bar graphs of the quantification of the effect of fenretinide on SK1a, p53 and p21 expression. Results are expressed as means +/− SD for *n* = 3 experiments. ***p < 0.001 *versus* control; (**B**) The effect of fenretinide on [^3^H]thymidine incorporation. Results are expressed as means +/− SD for *n* = 3 experiments.

We have previously reported that the treatment of prostate cancer cells with SKi reduces S1P levels and markedly increases sphingosine and dihydroceramide levels in these cells; the latter is consistent with inhibition of dihydroceramide desaturase, Des1 [4]. Indeed, we show here that ABC294640 or SKi inhibit Des1 activity in intact Jurkat cells (Figure 3). ABC294640 exhibits an IC_50_ = 10.2 +/− 3.7 μM (*n* = 3) and SKi exhibited an IC_50_ = 0.63–0.69 μM (*n* = 2). These were very similar with the IC_50_ values for inhibition of DNA synthesis of 19.3 μM and 1.29 μM for ABC294640 and SKi respectively in LNCaP-AI cells (Figure 2A). In addition, Cingolani et al. [19] have reported a K_i_ (0.3 μM) for inhibiton of Des1 by SKi. In addition, we show here that treatment of LNCaP-AI cells with ABC294640 induced a decrease in Des1 (Mr = 38 kDa) expression that was reversed by the proteasomal inhibitor, MG132 (Figure 3) indicating an additional level of regulation of Des1 by ABC294640. We therefore investigated whether the effects of ABC294640 and SKi on SK1a, p53 and p21 expression are mediated through inhibition of Des1 activity. We used the Des1 inhibitor, fenretinide [20] which induced a reduction in SK1a expression and an increase in p53 and p21 expression (Figure 4A). Fenretinide also induced a concentration-dependent inhibition of DNA synthesis in androgen-independent LNCaP-AI cells (Figure 4B).

### p53 and p21 expression but not proteasomal degradation of SK1 is regulated by an oxidative stress-dependent mechanism

Our findings suggest that Des1 activity normally functions to restrain proteasomal activity and that inhibition of the enzyme with SKi or ABC294640 results in the ubiquitin-proteasomal degradation of SK1a and Des1. In addition, Des1 appears to exert a regulatory influence on p53 and p21 expression. Therefore we next investigated whether SK1a, p53 and p21 are linked in a sequential linear or convergent parallel pathway to regulate growth of androgen-independent LNCaP-AI cells.

**Figure 5:**
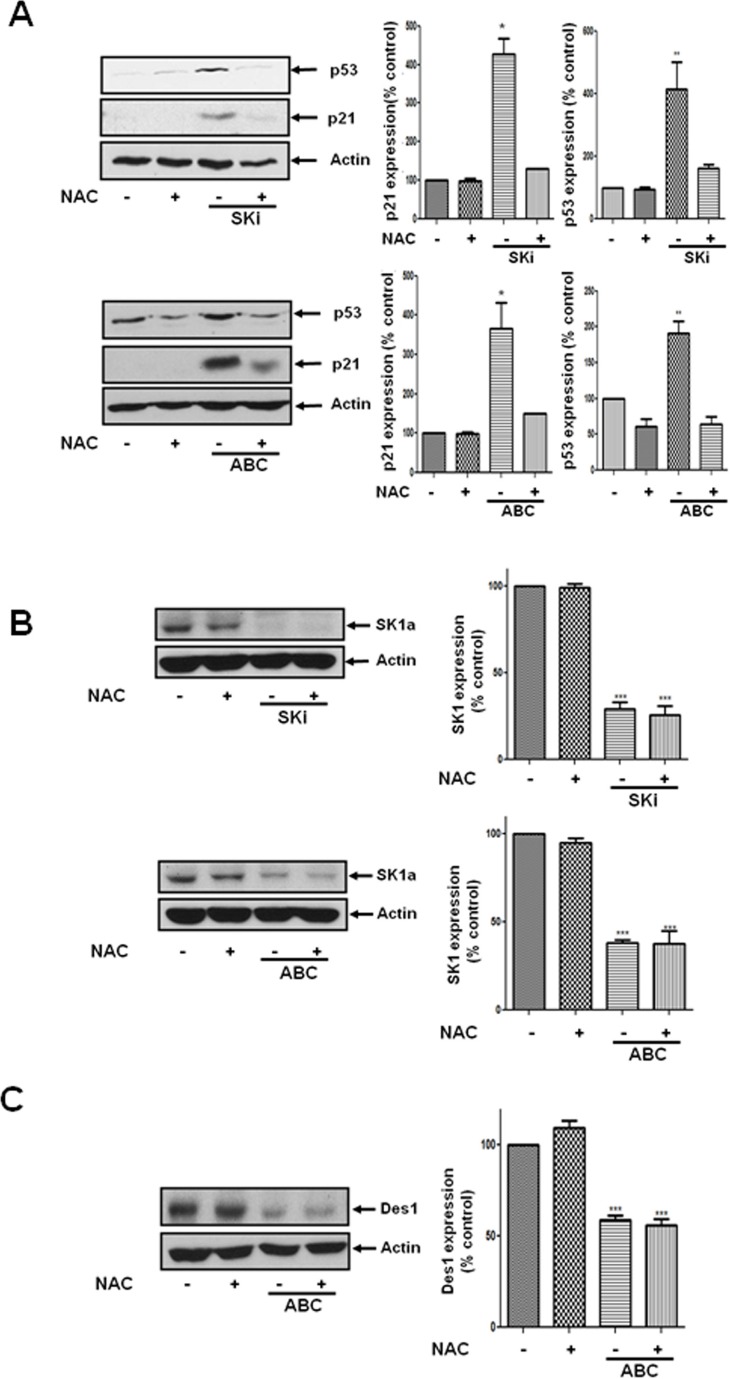
Effect of NAC on the ABC294640- or SKi-induced increase in p21 and p53 expression and the lack of effect on SK1a expression LNCaP-AI cells were pre-treated with or without NAC (10 mM) for 30 min prior to treatment with ABC294640 (25 μM) or SKi (10 μM) for 48 h. (**A**) Western blot showing the effect of NAC on the ABC294640- or SKi-induced increase in p53 and p21 expression; (**B**) Western blot showing the lack of effect of NAC on the ABC294640- or SKi-induced proteasomal degradation of SK1a; (**C**) Western blot showing the lack of effect of NAC on the ABC294640-induced proteasomal degradation of Des1. In (A-C) actin was used as a protein loading control. Western blot results are representative of at least three independent experiments. Also shown are bar graphs of the quantification of the effect of NAC (10 mM) on SK inhibitor induced in changes in p21, p53, SK1a and Des1 expression. Results are expressed as means +/− SD for *n* = 3 experiments. *p < 0.05 *versus* control, ***p < 0.001 *versus* control.

We have previously shown that SKi induces an oxidative stress response in androgen-independent LNCaP-AI cells as evidenced by the accumulation of oxidised glutathione and switching from aerobic glycolysis to the pentose phosphate pathway, which attempts to counteract oxidative stress by production of NADPH [15]. Moreover, Mercado et al. [21] demonstrated that SKi regulates Nrf2, a redox-sensitive transcription factor. Indeed, the effect of ABC294640 or SKi observed here on p53 and p21 expression was reversed by the reactive oxygen species scavenger, N-acetyl cysteine (Figure 5A). However, the effect of ABC294640 or SKi on the proteasomal degradation of SK1a was not modulated by N-acetyl cysteine (Figure 5B). In addition, N-acetyl cysteine had no effect on the ABC294640-induced proteasomal degradation of Des1 (Figure 5C), suggesting that the oxidative stress response is down stream of Des1.

**Figure 6:**
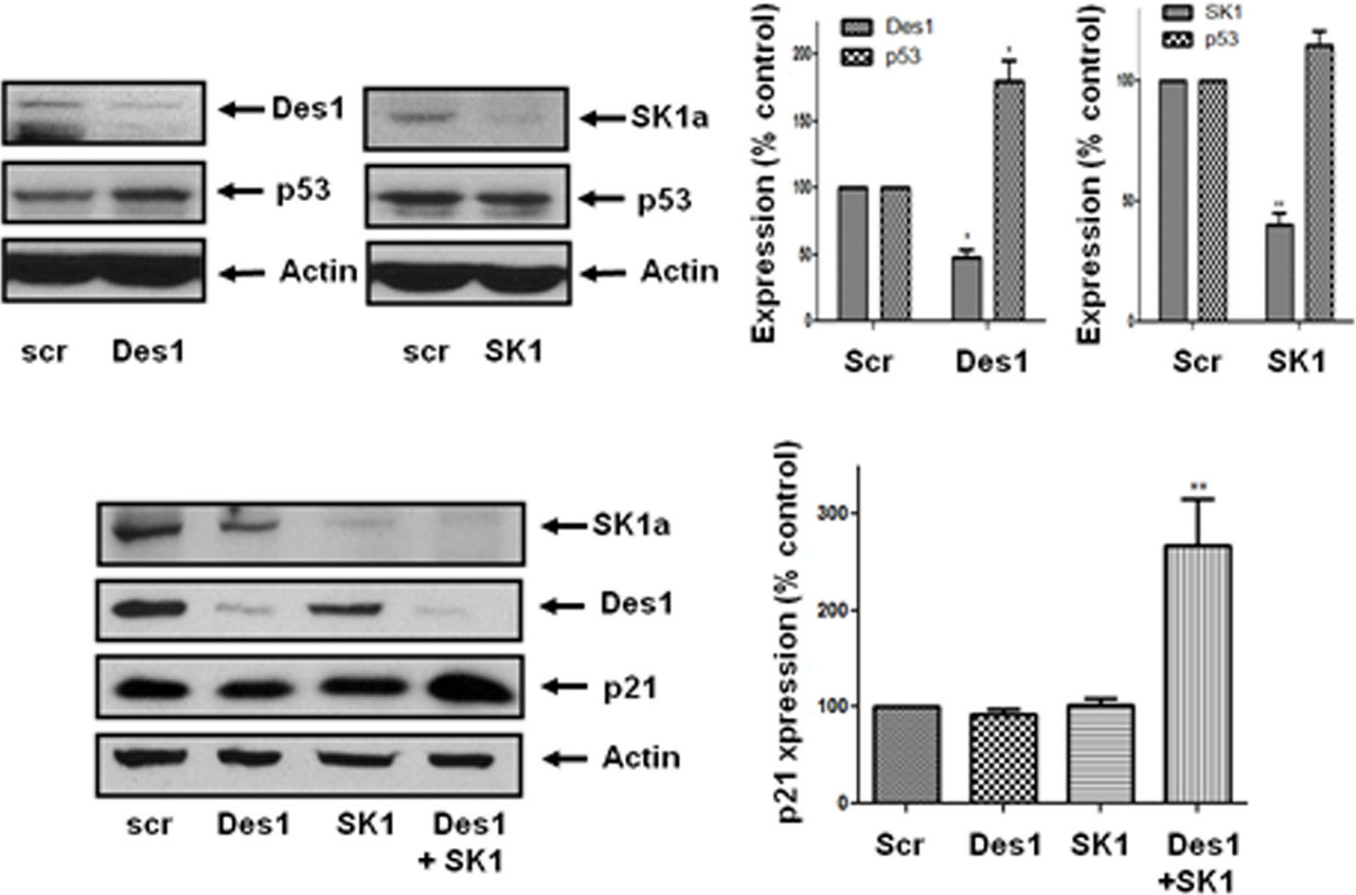
Effect of Des1 and SK1 siRNA on p21 and p53 expression in androgen-independent LNCaP-AI cells Androgen-independent LNCaP-AI cells were treated with scrambled siRNA or SK1 siRNA or Des1 siRNA for 48 h. Western blot showing the effect of SK1 siRNA or Des1 siRNA on SK1, Des1, p53 and p21 expression. Actin was used a protein loading control. Results are representative of at least three independent experiments. Also shown are bar graphs of the quantification of the effect of SK1 siRNA or Des1 siRNA on SK1a, Des1, p53 and p21 expression. Results are expressed as means +/− SD for *n* = 3 experiments. *p < 0.05, **p < 0.01 *versus* scrambled siRNA.

These findings place SK1a and p21/p53 on separate pathways. However, siRNA knockdown of Des1 increased p53 expression while a combination of Des1/SK1 siRNA increased expression of p21 (Figure 6). Therefore, p53 expression is regulated by Des1, while p21 is regulated by a combination of Des1 and SK1a. The Des1-dependent regulation of p21 and p53 appears to involve an oxidative stress response. Des1 activity has also been reported to be inhibited by oxidative stress [22].

## DISCUSSION

**Figure 7:**
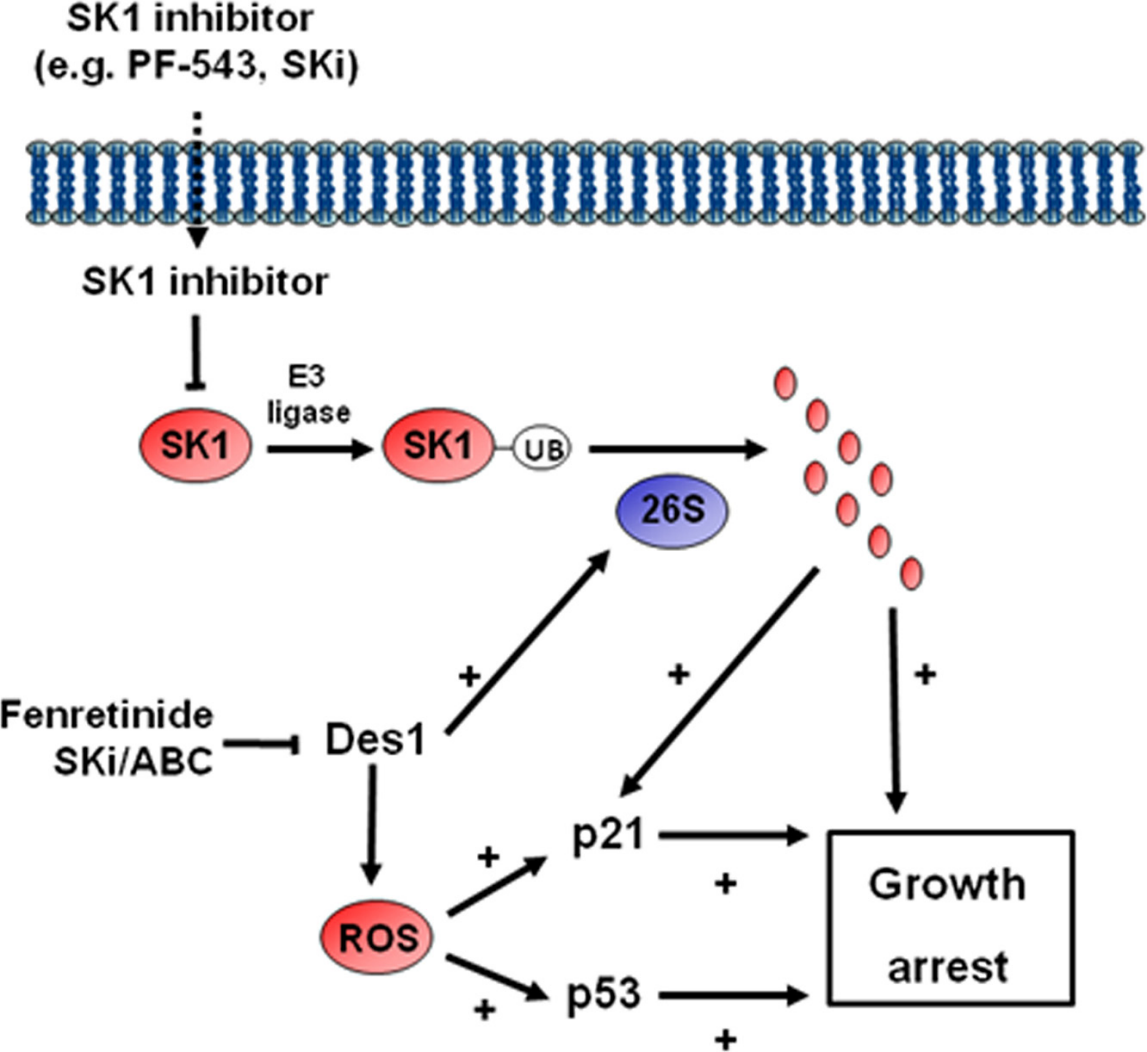
Schematic showing the mechanism by which ABC294640 or SKi induce growth arrest in androgen-independent LNCaP-AI cells

We have demonstrated that SK1 and Des1 participate in regulating growth of androgen-independent prostate cancer cells and this involves p53/p21-dependent and -independent pathways (Figure 7). This is supported by studies demonstrating that ABC294640 induces significant inhibition of growth, proliferation, and cell-cycle progression in prostate cancer cells [23]. In addition SKi induces growth arrest at S phase with a decrease in the proportion of A498 kidney adenocarcinoma cells in G2/M phase [24]. ABC294640 induced an arrest in G1 phase with a decrease in the proportion of A498 cells in G2/M and S phase [24]. These effects of SKi and ABC294640 might reflect different efficacies in terms of modulating SK1, SK2 and Des1 activities in A498 cells.

We have previously show that the treatment of LNCaP prostate cancer cells with SKi results in a substantial increase in the levels of several different molecular species of dihydroceramide [4] suggesting, in agreement with Cingolani et al. [19], that SKi inhibits Des1 activity. Indeed, we have confirmed here that both SKi and ABC294640 inhibit Des1 activity. Here, we establish that ABC294640 induces proteasomal degradation of Des1. Moreover, Venant et al. [25] have also demonstrated that ABC294640 reduces the growth of prostate cancer cells and inhibits Des1 activity and this is associated with an increase in dihydroceramide levels. Others have demonstrated that Des1 activity is inhibited by SKi in a non-competitive manner and that inhibition was indirect [19]. We propose here that Des1 functions to restrain proteasomal activity and that its inhibition by SKi or ABC294640, therefore, increases the proteasomal degradation of SK1a and Des1. Consistent with this, the dihydroceramide desaturase inhibitor, fenretinide fully recapitulated the effects of SKi and ABC294640 in promoting the proteasomal degradation of SK1a in androgen-independent LNCaP-AI prostate cancer cells.

The treatment of androgen-independent LNCaP-AI prostate cancer cells with SKi or ABC294640 or fenretinide also induced an increase in the expression of p53 and p21. In contrast, treatment of these prostate cancer cells with SK1 selective (PF-543) or SK2 selective (ROMe) inhibitors alone or siRNA knockdown of SK1 or SK2 or their combination failed to induce an increase in the expression of p53 and p21. Therefore, inhibition or knockdown of SK1 or SK2 alone is insufficient to affect p53/p21 expression. However, siRNA knockdown of Des1 increased p53 expression and a combination of Des1/SK1 siRNA-mediated knockdown induced an increased expression of p21. These findings suggest that the effects of SKi and ABC294640 on p53 expression are likely mediated by Des1 inhibition and this is supported by our finding that fenretinide increases p53 and p21 and reduces SK1 expression. In contrast, the increase in p21 expression by SKi or ABC294640 might involve regulation by both Des1 and SK1 (Figure 7). The SKi- and ABC294640-induced increase in p53 and p21 expression is also blocked by scavenging ROS with the anti-oxidant, N-acetyl cysteine, suggesting that the regulation of p53 and p21 by Des1 involves an oxidative stress response. In addition, siRNA knockdown of SK1 inhibited DNA synthesis, which was independent of p53 and p21 as these were not affected by SK1 siRNA alone. Therefore, growth arrest induced by SKi or ABC294640 appears to involve convergence of pathways regulated by SK1a and Des1. This is likely by modulation of the *de novo* ceramide pathway at Des1 (with regulation of p53 and p21) and proteasomal degradation of SK1 and Des1 (with regulation of p21) accompanied by non-senescence-dependent pathways, regulated by SK1, that impact the sphingolipid rheostat pathway (i.e. relative effects of ceramide and S1P).

The concept of targeting Des1 to promote cancer cell growth arrest is supported by several studies. For instance, Kraveka et al. [26] demonstrated that siRNA knockdown of Des1 in SMS-KCNR neuroblastoma cancer cells increased dihydroceramide levels and inhibited cell growth with promoted cell cycle arrest at G0/G1. This was associated with a protein phosphatase 1-induced dephosphorylation of retinoblastoma protein, which regulates cell cycle transition. In addition, Gagliostro et al. [27] have used the dihydroceramide desaturase inhibitor XM462 to increase dihydrocermaide levels and to induce autophagy in the gastric carcinoma HCG27 cell line. This was associated with modulation of cyclin D1 expression and delayed G1/S transition. These effects were recapitulated with dihydroceramide analogues, which caused cell cycle delay at the G1/S transition and promoted autophagy, which was linked with the cell cycle arrest.

Therefore, we propose that in order to reduce androgen-independent prostate cancer progression it is necessary to modulate both SK1/Des1 thereby affecting *de novo* ceramide and sphingolipid rheostat pathways. This concept suggests that targeting multiple stress responsive sphingolipid pathways with a single chemical entity is likely to be more effective than targeting a single pathway in terms of inducing growth arrest of cancer cells.

## MATERIALS AND METHODS

### Materials

All general biochemicals were from Sigma (Poole, UK). ABC294640 was from MedChemExpress (USA). SKi and PF543 were from Merck Biosciences (Nottingham, UK). RB-005 and F-02 were synthesized as described previously [7, 8]. MG132 was from Enzo Life Sciences (Exeter, UK). SK2 selective inhibitor (*R*)-FTY720 methylether (ROMe) was synthesized as described previously [16]. Antibodies were obtained as follows: anti-actin (#A2066), and anti-p53 (#P8999) from Sigma (Poole, UK); anti-myc (9E10) from Santa Cruz Biotechnology (Santa Cruz, CA), and anti-p21 (#2947) from New England Biolabs (Oxford, UK); anti-DES1 (EPR9680), antibody from Abcam; Anti-SK1 (lab reference number 48:2) antibody was custom made by Abcam using antigens detailed in [28]. DharmaFECT^™^ reagent, ON-TARGETplus SMARTpool^®^ SK1, SK2 and DES1 siRNAs were from Dharmacon (Cromlington, UK). Scrambled siRNA (ALLSTARS Negative control) was from Qiagen (Crawley, UK).

### Cell culture

LNCaP-AI (androgen-independent) cells and Jurkat T cells were maintained in RPMI supplemented with 1% (v/v) L-glutamine, 100 U/ml penicillin, 100 mg/ml streptomycin and 10% (v/v)lipid-stripped fetal calf serum or 10% (v/v) fetal bovine serum, respectively, at 37°C with 5% CO_2_.

### [^3^H] thymidine incorporation

LNCaP-AI cells (approx. 70% confluent) in 24 well plates were incubated with inhibitors or vehicle (DMSO, 0.1% v/v final), as detailed in the legends, for 20 h prior to the addition of [^3^H] thymidine (9.25 kBq per well) for a further 5 h. Incubations were terminated by removing the medium and immediately adding 1 ml of ice cold 10% (w/v) trichloroacetic acid and placed on ice for 10 minutes. This was replaced with a further 1 ml ice cold 10% (w/v) trichloroacetic acid for 10 minutes and repeated once more. Residual nuclear material was dissolved in 0.25 ml of 0.1% SDS/0.3 M NaOH. [^3^H] thymidine uptake was quantified by liquid-scintillation counting. Radiometric values (mean +/− SD) were obtained from 3 or more independent experiments.

### Western blotting

Upon treatment, LNCaP-AI cells were lysed in sample buffer (62.5 mM Tris-HCl (pH 6.7), 0.5 M sodium pyrophosphate, 1.25 mM EDTA, 1.25% (w/v) sodium dodecyl sulphate, 0.06% (w/v) bromophenol blue, 12.5% (v/v) glycerol (all from Sigma) and 50 mM dithiothreitol (Enzo)). Proteins were separated on a 10% (v/v) acrylamide/bisacrylamide gel, and transferred to nitrocellulose Hybond membrane (GE Healthcare). Membranes were blocked in 5% (w/v) BSA (Fisher) in TBST buffer (20 mM Tris-HCl (pH 7.5), 48 mM NaCl, 0.1% (v/v) Tween 20) for 1 hour at room temperature prior to incubation with primary antibody (diluted in blocking buffer) overnight at 4°C. Following three washes in TBST, membranes were incubated with horse radish peroxidase-conjugated anti-mouse or anti-rabbit IgG secondary antibody (Sigma; diluted in blocking buffer) for 1 hour at room temperature. Immunoreactive protein bands were visualized using enhanced chemiluminescence, acquired as TIFF files and quantified using ImageJ. Densitometric values (represented as mean +/− SD) were normalized using the corresponding data for actin for the same samples and were obtained from 3 or more independent experiments. Statistical analysis was undertaken using one way ANOVA with Tukey's post hoc test.

### siRNA transfections

LNCaP-AI cells were transiently transfected with siRNA constructs (as described in the figure legends) or scrambled siRNA (as a negative control) at a final concentration of 200 nM or 400 nM. Cells were grown to approximately 60% confluence and for each well a mixture of 4 μM siRNA in 50 μl of DNAse/RNAse free water and 1.6 μl of DharmaFECT^®^ 2 transfection reagent in 150 μl of serum free, antibiotic free media was prepared. These preparations were incubated for 5 minutes at room temperature to mix and then combined and incubated for 20 minutes at room temperature to allow formation of siRNA and DharmaFECT^®^ 2 transfection reagent complexes. Next, 800 μl of antibiotic-free RPMI 1640 medium supplemented with 10% (v/v) lipid-stripped fetal calf serum and 1% (v/v) L-glutamine were added to the transfection mix, which was then gently mixed. This was then used to replace cell culture medium and cells were incubated at 37°C in 5% CO_2_ for 48 hours.

### Des1 assays

Measurement of Des1 activity was performed by HPLC using intact Jurkat cells labeled with DhCer-C6-NBD as described previously [29]. Briefly, 10^6^ cells/ml Jurkat cells were incubated on ice for 30 minutes with 10 μM DhCer-C6-NBD (Cayman) in 0.5% serum in RPMI media. The cells were changed into fresh media and treated with inhibitors for 3 hours. The cells were harvested and pelleted via centrifugation at 500 *g* and resuspended in 100 ml of H_2_0. The samples were sonicated for 30 seconds followed by the addition of 900 ml methanol. Immediately before analysis, the samples were centrifuged for 3 mins at 13,000 *g* and transferred to HPLC vials. Extracted samples (50 μl) were analysed on a Waters HPLC coupled to a fluorescence detector using a 30 cm C18 reverse-phase column eluted with 1 ml/min 20% H_2_O and 80% acetonitrile with 0.1% trifluoroacetic acid. NBD-labelled substrate and product were quantitated with excitation and emission wavelengths of 465 nm and 530 nm, respectively.

## SUPPLEMENTARY MATERIALS FIGURE



## References

[1] Pyne NJ, Pyne S (2010). Sphingosine 1-phosphate and cancer. Nat Rev Cancer.

[2] Blaho VA, Hla T (2014). An update on the biology of sphingosine 1-phosphate receptors. J Lipid Res.

[3] Hait NC, Allegood J, Maceyka M, Strub GM, Harikumar KB, Singh SK, Luo C, Marmorstein R, Kordula T, Milstien S, Spiegel S (2009). Regulation of histone acetylation in the nucleus by sphingosine-1-phosphate. Science.

[4] Loveridge C, Tonelli F, Leclercq T, Lim KG, Long JS, Berdyshev E, Tate RJ, Natarajan V, Pitson SM, Pyne NJ, Pyne S (2010). The sphingosine kinase 1 inhibitor 2-(p-hydroxyanilino)-4-(p-chlorophenyl)thiazole induces proteasomal degradation of sphingosine kinase 1 in mammalian cells. J Biol Chem.

[5] Tonelli F, Lim KG, Loveridge C, Long J, Pitson SM, Tigyi G, Bittman R, Pyne S, Pyne NJ (2010). FTY720 and (S)-FTY720 vinylphosphonate inhibit sphingosine kinase 1 and promote its proteasomal degradation in human pulmonary artery smooth muscle breast cancer and androgen-independent prostate cancer cells. Cell Signal.

[6] Lim KG, Tonelli F, Li Z, Lu X, Bittman R, Pyne S, Pyne NJ (2011). FTY720 analogues as sphingosine kinase 1 inhibitors: enzyme inhibition kinetics, allosterism, proteasomal degradation, and actin rearrangement in MCF-7 breast cancer cells. J Biol Chem.

[7] Baek DJ, MacRitchie N, Pyne NJ, Pyne S, Bittman R (2013). Synthesis of selective inhibitors of sphingosine kinase 1. Chem Commun (Camb).

[8] Byun HS, Pyne S, Macritchie N, Pyne NJ, Bittman R (2013). Novel sphingosine-containing analogues selectively inhibit sphingosine kinase (SK) isozymes, induce SK1 proteasomal degradation and reduce DNA synthesis in human pulmonary arterial smooth muscle cells. Med Chem Comm.

[9] French KJ, Zhuang Y, Maines LW, Gao P, Wang W, Beljanski V, Upson JJ, Green CL, Keller SN, Smith CD (2010). Pharmacology and antitumor activity of ABC294640, a selective inhibitor of sphingosine kinase-2. J Pharmacol Exp Ther.

[10] Qin Z, Dai L, Trillo-Tinoco J, Senkal C, Wang W, Reske T, Bonstaff K, Del Valle L, Rodriguez P, Flemington E, Voelkel-Johnson C, Smith CD, Ogretmen B, Parsons C (2014). Targeting sphingosine kinase induces apoptosis and tumor regression for KSHV-associated primary effusion lymphoma. Mol Cancer Ther.

[11] Beljanski V, Knaak C, Smith CD (2010). A novel sphingosine kinase inhibitor induces autophagy in tumor cells. J Pharmacol Exp Ther.

[12] Xun C, Chen MB, Qi L, Tie-Ning Z, Peng X, Ning L, Zhi-Xiao C, Li-Wei W (2015). Targeting sphingosine kinase 2 (SphK2) by ABC294640 inhibits colorectal cancer cell growth *in vitro* and *in vivo*. J Exp Clin Cancer Res.

[13] Chumanevich AA, Poudyal D, Cui X, Davis T, Wood PA, Smith CD, Hofseth LJ (2010). Suppression of colitis-driven colon cancer in mice by a novel small molecule inhibitor of sphingosine kinase. Carcinogenesis.

[14] Venkata JK, An N, Stuart R, Costa LJ, Cai H, Coker W, Song JH, Gibbs K, Matson T, Garrett-Mayer E, Wan Z, Ogretmen B, Smith C (2014). Inhibition of sphingosine kinase 2 downregulates the expression of c-Myc and Mcl-1 and induces apoptosis in multiple myeloma. Blood.

[15] Watson DG, Tonelli F, Alossaimi M, Williamson L, Chan E, Gorshkova I, Berdyshev E, Bittman R, Pyne NJ, Pyne S (2013). The roles of sphingosine kinases 1 and 2 in regulating the Warburg effect in prostate cancer cells. Cell Signal.

[16] Lim KG, Sun C, Bittman R, Pyne NJ, Pyne S (2011). (R)-FTY720 methyl ether is a specific sphingosine kinase 2 inhibitor: Effect on sphingosine kinase 2 expression in HEK 293 cells and actin rearrangement and survival of MCF-7 breast cancer cells. Cell Signal.

[17] Schnüte ME, McReynolds MD, Kasten T, Yates M, Jerome G, Rains JW, Hall T, Chrencik J, Kraus M, Cronin CN, Saabye M, Highkin MK, Broadus R (2012). Modulation of cellular S1P levels with a novel, potent and specific inhibitor of sphingosine kinase-1. Biochem J.

[18] Ren S, Xin C, Pfeilschifter J, Huwiler A (2010). A novel mode of action of the putative sphingosine kinase inhibitor 2-(p-hydroxyanilino)-4-(p-chlorophenyl)thiazole (SKI II): induction of lysosomal sphingosine kinase 1 degradation. Cell Physiol Biochem.

[19] Cingolani F, Casasampere M, Sanllehí P, Casas J, Bujons J, Fabrias G (2014). Inhibition of dihydroceramide desaturase activity by the sphingosine kinase inhibitor SKI II. J Lipid Res.

[20] Rahmaniyan M, Curley RW, Obeid LM, Hannun YA, Kraveka JM (2011). Identification of dihydroceramide desaturase as a direct *in vitro* target for fenretinide. J Biol Chem.

[21] Mercado N, Kizawa Y, Ueda K, Xiong Y, Kimura G, Moses A, Curtis JM, Ito K, Barnes PJ (2014). Activation of transcription factor Nrf2 signalling by the sphingosine kinase inhibitor SKI-II is mediated by the formation of Keap1 dimers. PLoS One.

[22] Idkowiak-Baldys J, Apraiz A, Li L, Rahmaniyan M, Clarke CJ, Kraveka JM, Asumendi A, Hannun YA (2010). Dihydroceramide desaturase activity is modulated by oxidative stress. Biochem J.

[23] Schrecengost RS, Keller SN, Schiewer MJ, Knudsen KE, Smith CD (2015). Downregulation of Critical Oncogenes by the Selective SK2 Inhibitor ABC294640 Hinders Prostate Cancer Progression. Mol Cancer Res.

[24] Gao P, Peterson YK, Smith RA, Smith CD (2012). Characterization of isoenzyme-selective inhibitors of human sphingosine kinases. PLoS One.

[25] Venant H, Rahmaniyan M, Jones EE, Lu P, Lilly MB, Garrett-Mayer E, Drake RR, Kraveka JM, Smith CD, Voelkel-Johnson C (2015). The sphingosine kinase 2 inhibitor ABC294640 reduces the growth of prostate cancer cells and results in accumulation of dihydroceramides *in vitro* and *in vivo*. Mol Cancer Ther.

[26] Kraveka JM, Li L, Szulc ZM, Bielawski J, Ogretmen B, Hannun YA, Obeid LM, Bielawska A (2007). Involvement of dihydroceramide desaturase in cell cycle progression in human neuroblastoma cells. J Biol Chem.

[27] Gagliostro V, Casas J, Caretti A, Abad JL, Tagliavacca L, Ghidoni R, Fabrias G, Signorelli P (2012). Dihydroceramide delays cell cycle G1/S transition via activation of ER stress and induction of autophagy. Int J Biochem Cell Biol.

[28] Huwiler A, Döll F, Ren S, Klawitter S, Greening A, Römer I, Bubnova S, Reinsberg L, Pfeilschifter J (2006). Histamine increases sphingosine kinase-1 expression and activity in the human arterial endothelial cell line EA.hy 926 by a PKC-alpha-dependent mechanism. Biochim Biophys Acta.

[29] Pitman MR, Powell JA, Coolen C, Moretti PA, Zebol JR, Pham DH, Finnie JW, Don AS, Ebert LM, Bonder CS, Gliddon BL, Pitson SM (2015). A selective ATP-competitive sphingosine kinase inhibitor demonstrates anti-cancer properties. Oncotarget.

