# Supplementary material for: Proteasomal degradation of sphingosine kinase 1 and inhibition of dihydroceramide desaturase by the sphingosine kinase inhibitors, SKi or ABC294640, induces growth arrest in androgen-independent LNCaP-AI prostate cancer cells
Source: Oncotarget. 2016 Feb 25;7(13):16663–75. doi: 10.18632/oncotarget.7693 (PMC4941342; doi:10.18632/oncotarget.7693)
Supplement: Supplementary file 1 [file oncotarget-07-16663-s001.pdf]

Proteasomal degradation of sphingosine kinase 1 and inhibition of dihydroceramide desaturase by the sphingosine kinase inhibitors, SKi or ABC294640, induces growth arrest in androgen-independent LNCaP-AI prostate cancer cells

Supplementary Materials

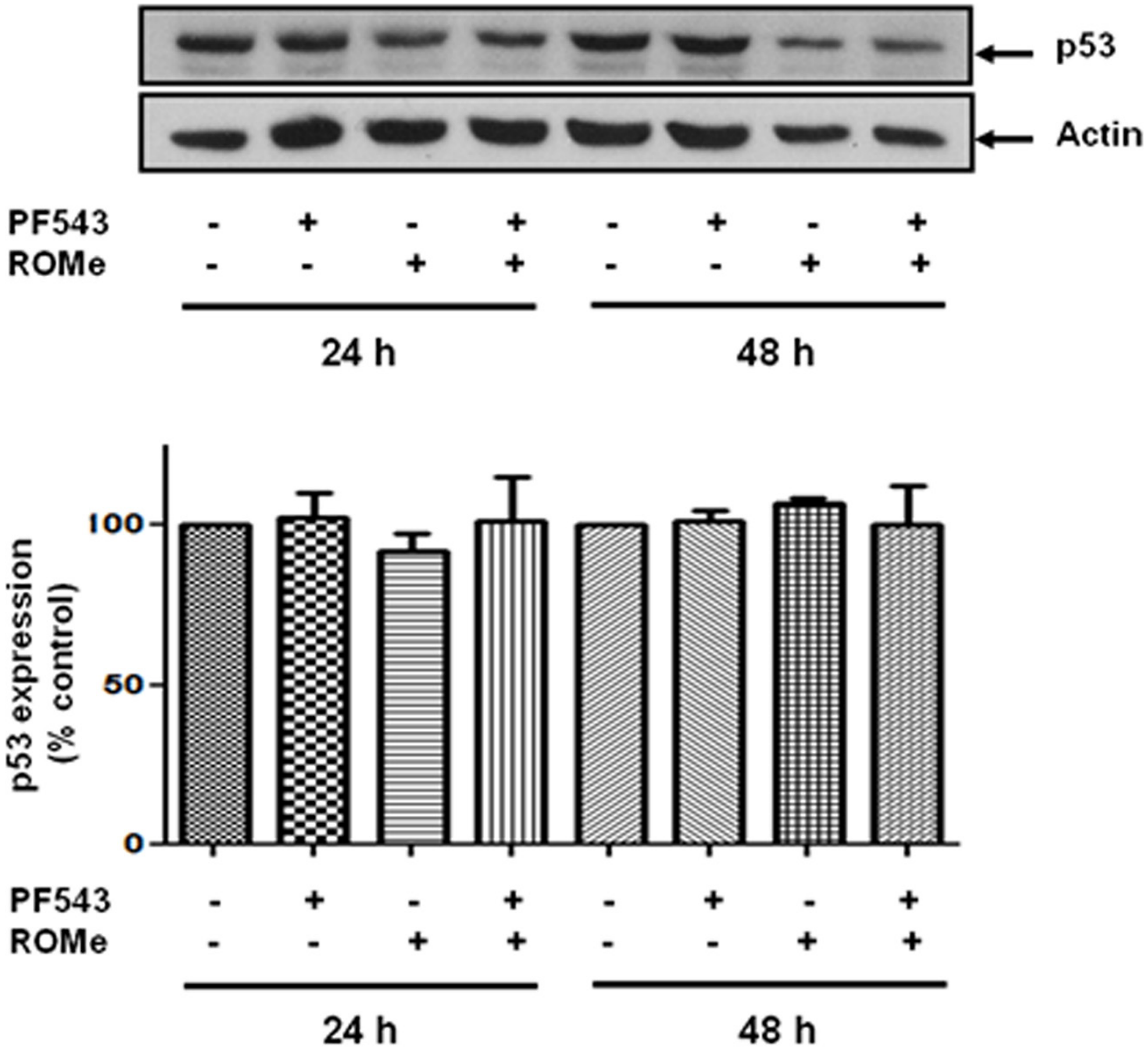

Supplementary Figure S1: Lack of effect of ROMe or PF-543 on p53 expression in androgen-independent LNCaP-AI cells. LNCaP-AI cells were treated with ROMe (10 µM) and/or PF-543 (100 nM) for 24 h or 48 h. Western blot showing the lack of effect of ROMe and PF-543 on p53 expression. Actin was used a protein loading control. Results are representative of at least three independent experiments. Also shown is a bar graph of the quantification of the lack of effect of PF-543 (100 nM) and ROMe (10 µM) on p53 expression. Results are expressed as means +/− SD for *n* = 3 experiments.
